# The relationship between alexithymia, depression, anxiety, and stress in elderly with multiple chronic conditions in China: a network analysis

**DOI:** 10.3389/fpsyt.2023.1209936

**Published:** 2023-07-17

**Authors:** Bin Shang, Ruirui Chen, Caifeng Luo, Fei Lv, Jing Wu, Xiao Shao, Qian Li

**Affiliations:** ^1^School of Medicine, Jiangsu University, Zhenjiang, China; ^2^Department of Nursing, Jingjiang College, Jiangsu University, Zhenjiang, China; ^3^University Hospital, Nanjing University of Aeronautics and Astronautics, Nanjing, China; ^4^Endoscopy Center, Suqian First People’s Hospital, Suqian, China; ^5^Department of Neurology, Suzhou Xiangcheng People’s Hospital, Suzhou, China

**Keywords:** multiple chronic conditions, alexithymia, depression, anxiety, stress, network analysis

## Abstract

**Objective:**

This study aimed to construct a network structure to investigate the connections between alexithymia, depression, anxiety, and stress in Chinese older adults with multiple chronic conditions (MCC), identifying core and bridge symptoms, and comparing the network structure across different levels of alexithymia.

**Methods:**

This study used a cross-sectional survey design and convenience sampling to recruit participants from six cities in Jiangsu Province. The study assessed the levels of alexithymia, depression, anxiety, and stress in older adults with MCC using the Toronto Alexithymia Scale (TAS-20) and the Depression Anxiety and Stress Scale-21 (DASS-21). Network analysis was performed using R language to identify core and bridge symptoms in the network and compare the network structure across different levels of alexithymia.

**Results:**

A total of 662 participants were included in the analysis, including 395 men and 267 women. The mean age was 70.37 ± 6.92 years. The finding revealed that the “Difficulty Identifying Feelings” (DIF) node had the highest strength centrality (strength = 2.49) and predictability (rp = 0.76) in the network. The next highest strength centrality was observed for “Meaningless” (strength = 1.50), “Agitated” (strength = 1.47), “Scared” (strength = 1.42), and “No look forward” (strength = 0.75). They were identified as core symptoms. The bridge strength analysis identified “Panic,” “Scared,” “No wind down,” “No initiative,” and “No positive” as the bridge symptoms. There were notable differences in the overall network structure and specific connections between the groups with and without alexithymia (*p* < 0.05).

**Conclusion:**

“DIF” is a core node in the network of older adults with MCC, indicating its significance as a potential target for psychological interventions in clinical practice. Preventing and mitigating bridge symptoms such as “panic,” “Scared,” “No wind down,” “No initiative,” and “No positive” can effectively impede the spread of symptom activation, thereby interrupting or severing the connections among comorbidities in older adults. Additionally, compared to non-alexithymia individuals, the psychological issues of older adults with alexithymia require prioritized intervention from healthcare professionals.

## 1. Introduction

The aging population and the prevalence of chronic diseases in the elderly have become a significant public health issue globally (1). In China, around 180 million elderly people suffer from chronic diseases, accounting for 75% of the elderly population (2). Moreover, the prevalence of multiple chronic conditions (MCC) in the elderly is as high as 65.14% (3). Older adults with MCC not only face physical discomfort and ongoing treatment costs but also commonly experience psychological problems such as depression, anxiety, and stress (4). Studies have shown that these negative psychological symptoms interact with each other and often co-occur in older adults with MCC (5). It is crucial to understand the psychological well-being of older adults with MCC, intervene in their psychological health, and facilitate their rehabilitation and treatment. Vasiliadis et al. highlighted the link between psychological distress and physical burden in older adults, emphasizing the impact of disease burden (6). Su et al. demonstrated the influence of psychological problems on subjective cognitive functioning and the role of self-efficacy in older adults (7). Wei et al. showed the interconnectedness of depression and anxiety, often co-occurring and influencing individuals (8). However, most studies on psychological problems have focused on foreign populations, and the psychological distress experienced by older adults may vary across countries due to cultural and geographical differences. Further research is needed to explore psychological distress in Chinese older adults, particularly those with MCC. Moreover, limited research has examined the impact of alexithymia on psychological distress in older adults (9, 10), especially those with MCC. Nevertheless, it is evident that older adults with MCC are more susceptible to these emotional issues (11, 12).

Alexithymia, initially described by Sifneos (13), is a cognitive-affective disorder characterized by difficulties in processing, regulating, and recognizing emotions. It is prevalent among elderly patients with chronic diseases (14, 15), with a global prevalence ranging from 10 to 60% (16). Elderly patients with MCC have a higher prevalence of alexithymia compared to those with a single chronic condition, and the prevalence increases with age (17). Alexithymia affects communication skills, disease management, recovery, and overall quality of life and mental health in the elderly. Studies have shown that individuals with alexithymia are more susceptible to anxiety, depression, and suicidal tendencies (18). Physical symptoms often accompany alexithymia, further impacting patients’ recovery and treatment outcomes (19). Additionally, there is a detrimental cycle between anxiety, depression, and alexithymia (18). Although research (9, 10) on the relationship between alexithymia and psychological distress has mainly focused on adolescents, it is essential to investigate the significant role of alexithymia in psychological distress among older adults with MCC. Understanding this role can contribute to promoting the mental and physical well-being of elderly individuals in later life.

Previous studies have primarily used the common cause theory (20) and latent variable perspectives (21) to explore the link between alexithymia and psychological issues. These approaches focus on singular influences between symptoms, assuming the presence of a latent common cause or underlying variables. They often use total scores on scales or statistical techniques like structural equation modeling, Pearson correlation analysis, or multiple regression analysis to assess the severity and correlation of psychological problems (21). However, these approaches overlook the distinct individual symptoms within anxiety and depression. Symptoms such as meaninglessness, worthlessness, sadness, worry, and panic interact in complex ways, contributing to the development of anxiety and depression. Some symptoms act as primary indicators, while others serve as bridges connecting with other disorders. It is crucial to identify and intervene in these specific symptoms for significant outcomes. Traditional methods like correlation analysis and multiple regression analysis may not fully capture the relationships between specific dimensions of alexithymia and individual symptoms of depression, anxiety, and stress (20, 21). Alternative approaches are needed to comprehensively understand the intricate associations and interactions among specific symptoms and their relationship with alexithymia and psychological distress.

The symptom network approach is a valuable tool for understanding the intricate interactions between alexithymia and psychological distress, such as depression, anxiety, and stress (22). It involves characterizing the collective symptoms related to a patient’s condition and quantitatively studying the associations between these symptoms using complex network analysis (22). This method applies network analysis principles, treating symptoms as nodes and their relationships as edges to construct a symptom network (23). By assessing the centrality of nodes, core symptoms within the network can be identified (24). These core symptoms have the highest activation and closely interact with other symptoms in the network. Targeting these core symptoms with psychological interventions can effectively weaken the network connections and improve intervention efficiency. Bridge symptoms within the network can be identified by assessing the bridge centrality index of a node (25). These symptoms have the strongest connections between symptom clusters. Interventions targeting bridge symptoms can sever the associations between these clusters. The study by Blanke et al. employed symptom network analysis to investigate the direct effects of cognitive-behavioral therapy on insomnia and its indirect effects on depression (26). Their findings provide valuable guidance for clinical practitioners aiming to alleviate symptoms of insomnia and depression effectively. Similarly, Zhu et al. utilized network analysis to compare core symptom differences among HIV patients with varying durations of illness, offering specific guidance for implementing precise intervention measures (27). Overall, the symptom network approach provides a comprehensive understanding of symptom interactions and serves as a valuable tool for guiding interventions in psychological research and clinical practice.

Therefore, this study aimed to use symptom network analysis to explore the relationship between alexithymia and depression, anxiety, and stress in elderly patients with MCC. The goal is to identify core and bridge symptoms in the network and analyze the mechanisms that influence their connection. Additionally, the study will classify alexithymia based on its severity and construct separate symptom networks to compare the characteristics and differences in network structures among different alexithymia groups for depression, anxiety, and stress. By thoroughly investigating the mechanisms underlying psychological problems in elderly patients with MCC, the study aims to provide valuable theoretical and practical support for improving their mental health. Based on our review of prior literature, this is the first study in Chinese communities that employs a symptom network analysis approach to examine the relationship between alexithymia and depression, anxiety, and stress in older adults with MCC.

## 2. Materials and methods

### 2.1. Study design and participants

This cross-sectional study involving multiple geographic areas was conducted, primarily using a convenience sampling method, between November 2022 and March 2023. According to the requirements for network analysis sample size (28), the sample size should be larger than the total parameters (including threshold parameters and pairwise correlation parameters). The threshold parameter equals the number of nodes, and the pairwise correlation parameter equals (total number of nodes) × (total number of nodes − 1) / 2. In this study, a total of 24 nodes needs to be constructed, therefore the threshold parameter is 24, and the pairwise correlation parameter is (24 × 23) / 2 = 276. Hence, the minimum sample size required is 300 participants. The sample was drawn from older adults with MCC in communities and villages in six prefecture-level cities (Nanjing, Suzhou, Changzhou, Zhenjiang, Lianyungang, and Suqian) in Jiangsu Province, China. The inclusion criteria were as per the following: (1) age ≥60 years; (2) suffering from two or more chronic diseases; and (3) informed consent and voluntary participation in this study. The exclusion criteria were as so: (1) elderly people who declined to participate in the study; (2) those with cognitive dysfunction or intellectual disabilities that may affect the validity of this study; (3) non-residents or residents who could not identify their place of residence; and (4) those involved in other research projects. This study protocol adheres to the guidelines detailed in the Declaration of Helsinki and was reviewed by the Medical Ethics Committee of Jiangsu University, under approval number 20221019-7.

Our data collection used a combination of online and offline methods. Previous research experience has taught us that using multiple data collection methods can better avoid missing key samples (29, 30). The online component used an online survey platform, Questionnaire Star,^1^ to distribute the questionnaire, and a uniformly trained researcher from the team used consistent instructions to distribute the questionnaire to eligible respondents and to explain the purpose, meaning, completion, and precautions of the survey. The survey was conducted using voluntary participation and anonymity. All participants agreed before filling out the questionnaire and, if they refused to participate, they could voluntarily exit the link. If they chose the latter, their responses would not be recorded. For some elderly participants who were illiterate or found the online questionnaire challenging to complete, the researcher asked questions verbally and filled in the online questionnaire with their answers. Additionally, to account for elderly participants who did not have access to smartphones, we prepared a paper questionnaire identical to the online questionnaire and followed the same completion requirements.

### 2.2. Measurements

#### 2.2.1. Demographic information and disease characteristics

Demographic and disease-related information collected in this study included the following: gender, age, marital status, place of residence, residential status, per capita monthly household income, number of chronic diseases, and type of medication taken.

#### 2.2.2. The Toronto Alexithymia Scale

We used the Chinese version of the Toronto Alexithymia Scale (TAS-20) to measure the degree of alexithymia. The scale was developed by Taylor (31) and later formed into a Chinese version after cross-cultural debugging by Jin et al. (32). The Cronbach’s alpha coefficient of the Chinese version of the scale is 0.830. There were 20 items in total, containing three dimensions; namely, difficulty identifying feelings (DIF), difficulty describing feelings (DDF), and externally oriented thoughts (EOTS). A Likert 5-point scale was used; 1 (completely disagree) to 5 (completely agree), where items 4, 5, 10, 18, and 19 were scored in reverse, with a total score of 20–100. Higher scores represent higher levels of alexithymia in the individuals. A total score <51 indicates no alexithymia, a total score between 52 and 60 indicates suspected alexithymia and a total score ≥61 can be judged as having alexithymia. In China, this scale has been shown to be a good tool for measuring alexithymia (33). In the current study, the Cronbach’s alpha coefficient for the total scale was 0.890, the Cronbach’s alpha coefficient for the three dimensions DIF, DDF, and EOTS are 0.907, 0.754, and 0.456. The EOTS dimension should be interpreted with caution.

#### 2.2.3. The Depression Anxiety and Stress Scale-21

We used a simplified version of the Depression Anxiety and Stress Scale-21 (DASS-21) to measure depression, anxiety, and stress levels in older adults with MCC. The scale is a simplified version based on the DASS scale developed by Lovibond and Lovibond (34), which was later revised by Gong et al. (35) to form a simplified Chinese version. The full scale contains a total of 21 entries, and the three subscales of depression, anxiety, and stress each contains 7 entries, all of which are rated on a 4-point Likert scale—0 (does not meet) to 3 (always meets)—with higher total scores representing the presence of higher negative emotions. The DASS-21 has been widely used in different countries and samples with good reliability and validity (36). In the current study, Cronbach’s alpha coefficient for the total scale was 0.943, and Cronbach’s alpha coefficients for the three dimensions of depression, anxiety, and stress were 0.874, 0.830, and 0.844, respectively.

### 2.3. Statistical analysis

We used SPSS version 26.0 for the descriptive statistics and different R packages from R version 4.2.0 for network analysis with the aim of exploring the relationship between alexithymia and depression, anxiety, and stress in older adults with MCC in Chinese communities. In the network, Items that are reverse scored (including items 4, 5, 10, 18, and 19) are converted into positive scores, and the three dimensions of alexithymia (DIF, DDF, and EOTS) were used as 3 nodes. The 21 entries in the Depression Anxiety and Stress Scale-21 were used as 21 nodes, for a total of 24 nodes in the final drawn network. We conducted four main analyses: network estimation (23), centrality and predictability measures (37), accuracy and stability estimation (28), and network comparison (38). All network visualizations were presented using the Fruchterman–Reingold algorithm in the qgraph package (39).

#### 2.3.1. Estimated network

We utilized the qgraph package (version 1.9.2) and the bootnet package (version 1.5.0) in R to construct a network representing the interactions between alexithymia, depression, anxiety, and stress in older adults (28). For network estimation, we employed the graph least absolute shrinkage and selection operator (gLASSO) method (40) and the extended Bayesian information criterion (EBIC) (41). These methods allowed us to minimize spurious connections and obtain a more realistic network structure (28). The gamma parameter in EBIC determined the reduction of spurious edges, and we set it to the default value of 0.5, as recommended in the literature (28). In the network representation, each symptom, such as “Meaningless,” was considered a node, and the line connecting neighboring symptoms represented an edge, with thicker edges indicating stronger correlations between the nodes. The color of the edges indicated the direction of correlation, with blue representing positive correlation and red representing negative correlation (23). To visualize the network, we employed the Fruchterman–Reingold algorithm, which positioned the core nodes closer to the center and the edge nodes on the periphery of the network (39).

#### 2.3.2. Centrality and predictability measurement

To evaluate and quantify the significance of each node (symptom) in the network, we computed three key centrality indices: strength, closeness, and betweenness (28). However, prior research (42) has demonstrated that closeness and betweenness are not reliable measures in mental health-related network analyses. Therefore, this study primarily focused on strength, the most commonly used centrality metric. Strength represents the sum of weighted connections of a node and measures its importance in the network. A higher strength value for a node (symptom) indicates a stronger connection and a more influential role in the network of older individuals, thus identifying it as a core symptom. Furthermore, we utilized the networktools package (version 1.5.0) to estimate the bridge centrality index, aiming to identify the bridge symptoms that connect the four conditions of alexithymia, depression, anxiety, and stress (25). Bridge symptoms refer to the symptoms that link different symptom clusters and reflect the degree of connectivity between the current node and other cluster nodes. Additionally, we employed the mgm package (version 1.2-12) to estimate the predictability of each node. Predictability (43) quantifies the extent to which changes in a node can be explained by changes in its connected nodes and reflects the controllability of the network. Higher predictability values indicate a greater influence of neighboring symptoms on a particular symptom in older adults with MCC (44). We denote predictability as rp.

#### 2.3.3. Estimating the accuracy and stability of the network

We validated the accuracy and stability of the constructed network using the R package bootnet (version 1.5.0) (28). Accuracy was assessed by calculating 95% confidence intervals (CIs) for the edge weights. We used a non-parametric bootstrap method (1,000 bootstrap samples) to construct the CIs, and fewer overlaps in the 95% CIs represented more accurate edge estimates. Stability assessment was performed by calculating the correlation stability coefficient of the strength centrality using the case-dropping subset bootstrap (1,000 bootstrap samples). That is, the network is considered stable if the centrality of the nodes does not change significantly after most of the samples are excluded from the dataset. The correlation stability coefficient should preferably be greater than 0.5, but at least greater than 0.25, and it is optimal if it is greater than 0.7 or more (28). We used rcS to denote the correlation stability coefficient. Finally, we performed bootstrap variability tests on the edge weights and node strengths (1,000 bootstrap samples, α = 0.05) to assess whether there is a significant difference between two edge weights or two node strengths.

#### 2.3.4. Network comparison

To examine potential differences in the depression, anxiety, and stress networks among different levels of alexithymia, we conducted a Network Comparison Test (NCT) using the R package NetworkComparisonTest (version 2.2.1) to assess network differences (38). The NCT is a two-tailed substitution method for comparing two networks, considering network structure invariance, global strength invariance, and edge strength invariance. Global strength invariance and edge strength invariance tests are performed only if network structural invariance is violated. In our study, as we observed discrepancies in the network structure, we conducted tests for global strength invariance and edge strength invariance. Furthermore, we employed the Holm-Bonferroni correction to conduct multiple comparisons of different edge strengths, aiming to identify specific edges that differed significantly between the two networks. A significance level of *p* < 0.05 was used to determine the presence of a significant difference between the networks.

## 3. Results

### 3.1. General characteristics of participants

We eventually included 662 eligible participants, including 395 (59.7%) men and 267 (40.3%) women. The mean age of the elderly with MCC was 70.37 years. Most of the older adults were married (89.6%), resided in rural areas (56.6%), and lived predominantly with their spouses (58.2%), as is detailed in Table 1. The mean scores for each scale entry are detailed in Table 2.

**TABLE 1:** General characteristics of co-morbid elderly.

Variables	*N*/% or Mean ± SD
**Gender**	
Male	395 (59.7)
Female	267 (40.3)
**Age**	
Age	70.37 (6.92)
**Marital status**	
Single	11 (1.7)
Married	593 (89.6)
Widowed	58 (8.8)
**Residence**	
Rural	375 (56.6)
Urban	287 (43.4)
**Residence status**	
Living alone	119 (18.0)
Living with spouse	385 (58.2)
Living with children	158 (23.9)
**Average monthly household income**	
3,000 CNY and below	259 (39.1)
3,000–5,000 CNY	240 (36.3)
5,000–8,000 CNY	132 (19.9)
8,000 CNY and above	31 (4.7)
**Number of chronic diseases**	
2 kinds	317 (47.9)
3 kinds	264 (39.9)
4 kinds and above	81 (12.2)
**Type of medication taken**	
None	23 (3.5)
1–2 kinds	272 (41.1)
3–4 kinds	248 (37.5)
5 kinds and above	119 (18.0)

**TABLE 2:** Content and mean scores of items on the TAS-20 and DASS-21.

Items	Abbreviations	Mean	SD
D1 I found it hard to wind down	No wind down	0.83	0.73
D2 I was aware of dryness of my mouth	Dry mouth	0.98	0.77
D3 I couldn’t seem to experience any positive feeling at all	No positive	0.75	0.75
D4 I experienced breathing difficulty	Breath difficult	0.67	0.73
D5 I found it difficult to work up the initiative to do things	No initiative	0.73	0.75
D6 I tended to over-react to situations	Over-react	0.89	0.74
D7 I experienced trembling (e.g., in the hands)	Trembling	0.82	0.79
D8 I felt that I was using a lot of nervous energy	Nervous energy	1.10	0.77
D9 I was worried about situations in which I might panic and make a fool of myself	Worried	0.77	0.77
D10 I felt that I had nothing to look forward to	No look forward	0.86	0.80
D11 I found myself getting agitated	Agitated	0.77	0.75
D12 I found it difficult to relax	No relax	0.94	0.74
D13 I felt down-hearted and blue	Down-hearted	0.90	0.79
D14 I was intolerant of anything that kept me from getting on with what I was doing	Intolerant	0.67	0.72
D15 I felt I was close to panic	Panic	0.68	0.75
D16 I was unable to become enthusiastic about anything	Not enthusiastic	0.89	0.73
D17 I felt I wasn’t worth much as a person	Worthless	0.49	0.69
D18 I felt that I was rather touchy	Touchy	0.76	0.72
D19 I was aware of the action of my heart in the absence of physical exertion	Heart aware	0.85	0.75
D20 I felt scared without any good reason	Scared	0.91	0.76
D21 I felt that life was meaningless	Meaningless	0.77	0.77
S1 Difficulty identifying feelings	DIF	19.61	5.06
B1 Difficulty describing feelings	DDF	14.36	3.12
W1 Externally oriented thoughts	EOTS	22.57	3.09

### 3.2. Network structure, centrality, and predictability measurements

**FIGURE 1:**
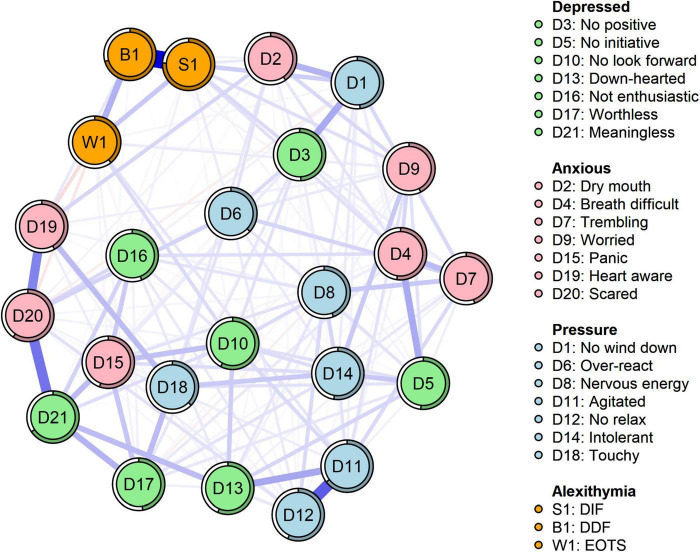
Network structure of alexithymia, depression, anxiety, and stress (*N* = 662). The same colors represent the same symptom clusters, where green represents depression, pink represents anxiety, blue represents stress, and orange represents alexithymia. DIF, difficulty identifying feelings; DDF, difficulty describing feelings; EOTS, externally oriented thoughts.

**FIGURE 2:**
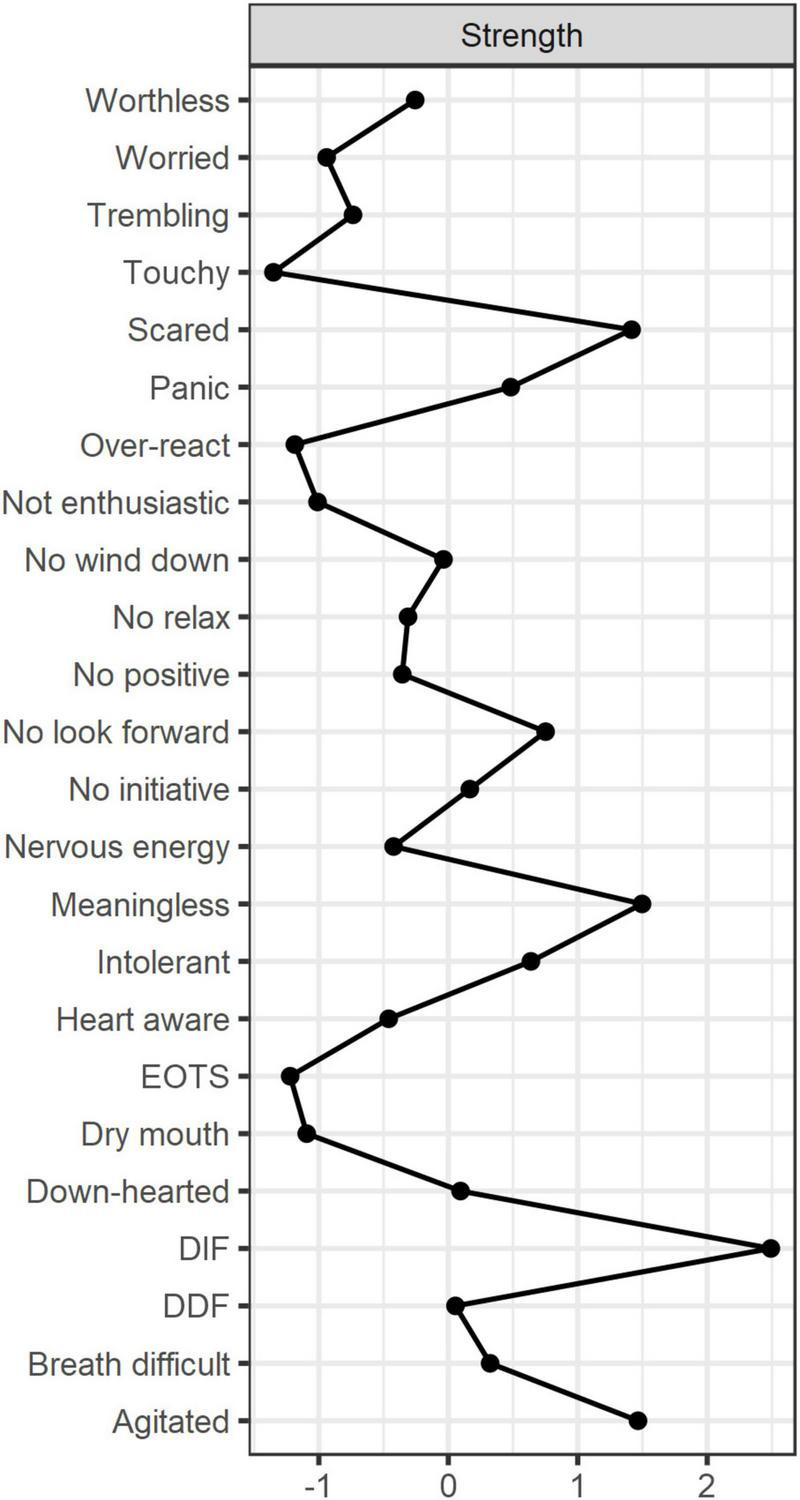
Centrality diagram of the network structure. Strength measures the importance of the nodes. DIF, difficulty identifying feelings; DDF, difficulty describing feelings; EOTS, externally oriented thoughts.

Figure 1 displays the network structure map between alexithymia and symptoms of depression, anxiety, and stress in 662 co-morbid older adults, which was constructed based on the EBICglasso model. Out of the 276 possible edges, 175 (63%) were non-zero edges, and the majority of edges showed positive correlations. The predictability of symptoms is presented in Figure 1 and Table 3 using a circular pie chart. The mean predictability value of symptoms in older adults with MCC was 0.51. Figure 2 presents the centrality indices of the network: strength, closeness, betweenness, and expected impact. In our constructed network containing 24 items, we found that “DIF” and “DDF” (weight = 0.64), “Agitated” and “No relax” (D11–D12, weight = 0.41), “Scared” and “Meaningless” (D20–D21, weight = 0.35), “Heart aware” and “Scared” (D19–D20, weight = 0.31), “Agitated” and “Down-hearted” (D11–D13, weight = 0.22) had a strong edge between them. The edge weight values between symptoms are shown in Supplementary Table S1. Figure 2 and Table 2 display the graphs and specific values of the centrality measures of the network, respectively. As we mentioned in the previous section, we used strength as the main centrality measure. We found that the top five symptoms with the highest strength in the constructed network structure were “DIF” (strength = 2.49), “Meaningless” (strength = 1.50), “Agitated” (strength = 1.50), “Scared” (strength = 1.42), and “No look forward” (strength = 0.75). The top five symptoms with the highest predictability were “DIF” (rp = 0.76), “DDF” (rp = 0.71), “Meaningless” (rp = 0.66), “Agitated” (rp = 0.65), and “Scared” (rp = 0.60).

**TABLE 3:** Centrality, bridge centrality, and predictability indices for the alexithymia, depression, anxiety, and stress scales.

Symptoms	Predictability	Strength	Bridge strength
D1 No wind down	0.49	−0.04	0.80
D2 Dry mouth	0.40	−1.09	0.52
D3 No positive	0.49	−0.36	0.70
D4 Breath difficult	0.52	0.32	0.66
D5 No initiative	0.52	0.17	0.72
D6 Over-react	0.37	−1.19	0.60
D7 Trembling	0.45	−0.74	0.67
D8 Nervous energy	0.44	−0.42	0.63
D9 Worried	0.43	−0.94	0.58
D10 No look forward	0.58	0.75	0.66
D11 Agitated	0.64	1.47	0.67
D12 No relax	0.57	−0.31	0.35
D13 Down-hearted	0.56	0.09	0.48
D14 Intolerant	0.53	0.64	0.65
D15 Panic	0.56	0.48	0.96
D16 Not enthusiastic	0.44	−1.01	0.55
D17 Worthless	0.47	−0.26	0.55
D18 Touchy	0.37	−1.35	0.54
D19 Heart aware	0.42	−0.46	0.41
D20 Scared	0.60	1.42	0.82
D21 Meaningless	0.65	1.50	0.60
S1 DIF	0.76	2.49	0.56
B1 DDF	0.71	0.06	0.14
W1 EOTS	0.39	−1.22	0.46

### 3.3. Network accuracy and stability

**FIGURE 3:**
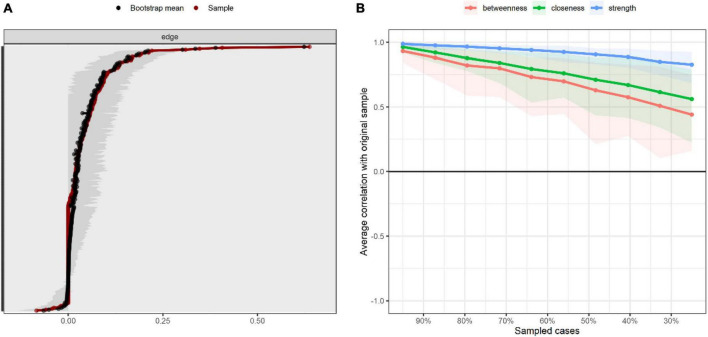
Accuracy and stability of network structure. **(A)** Accuracy analysis of edge weights. **(B)** Stability analysis of centrality indicators.

**FIGURE 4:**
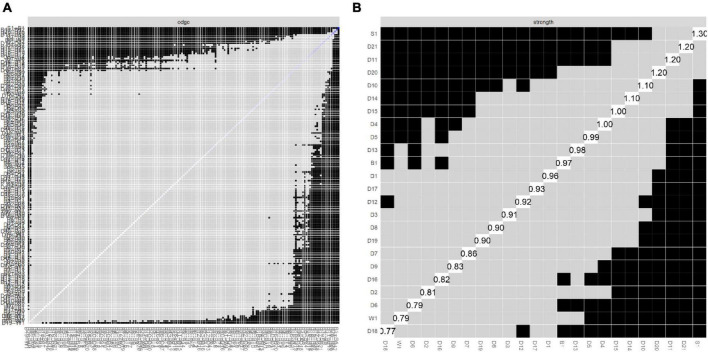
Bootstrapped difference tests for edge and strength. **(A)** Bootstrapped discrepancy test for edge weighting. **(B)** Bootstrapped discrepancy test for strength centrality. D1, no wind down; D2, dry mouth; D3, no positive; D4, breath difficult; D5, no initiative; D6, over-react; D7, trembling; D8, nervous energy; D9, worried; D10, no look forward; D11, agitated; D12, no relax; D13, down-hearted; D14, intolerant; D15, panic; D16, not enthusiastic; D17, worthless; D18, touchy; D19, heart aware; D20, scared; D21, meaningless; S1, DIF; B1, DDF; W1, EOTS.

We tested the accuracy and stability of the network structure by estimating 95% CIs for the edge weights. After estimation, we derived a central stability coefficient rcS = 0.75 > 0.70, which indicates that our results were sufficiently stable. The test of the variability of the edge weights showed that most of the edges were statistically significant (*p* < 0.05) (see Figures 3 and 4).

### 3.4. Bridge symptoms in the network

**FIGURE 5:**
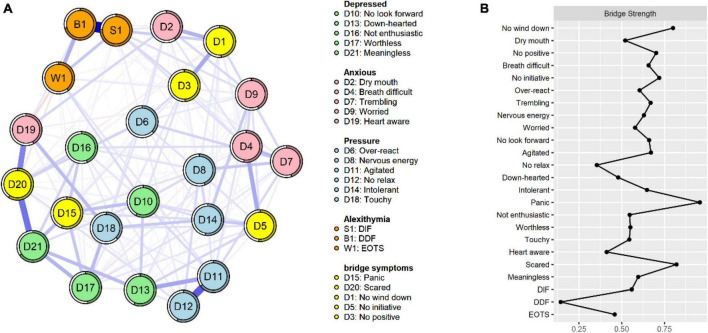
**(A)** Network structure diagram containing bridge symptoms. **(B)** Centrality index of bridge symptoms.

As shown in Figure 5 and Table 2, we found that “panic” (D15, bridge strength = 0.96), “scared” (D20, bridge strength = 0.82), “No wind down” (D1, bridge strength = 0.80), “No initiative” (D5, bridge strength = 0.72), and “No positive” (D3, bridge strength = 0.70) had the highest bridge strength, indicating that they are bridge symptoms in the network of alexithymia, depression, anxiety, and stress. According to the edge weighting relationship we found, the symptom “panic” (D15), which has the strongest bridge strength, was strongly related to the depression clusters “Meaningless” (D15–D21, weight = 0.17), “No look forward” (D15–D10, weight = 0.16), and “Worthless” (D15–D17, weight = 0.14). The bridge symptom “Scared” (D20) was strongly associated with “Meaningless” (D20–D21, weight = 0.35) in the depression cluster and “Over-react” (D20–D6, weight = 0.11) in the stress cluster. The bridge symptom “No wind down” (D1) was strongly correlated with “No positive” (D1–D3, weight = 0.21) in the depression cluster, “Dry mouth” (D1–D2, weight = 0.19) in the depression cluster, “Dry” (D1–D2, weight = 0.19) in the anxiety cluster, and “DIF” (D1–S1, weight = 0.19) in the alexithymia cluster. The bridge symptom “No initiative” (D5) was strongly associated with “Breath difficult” (D5–D4, weight = 0.21) in the anxiety cluster, “Intolerant” (D5–D14, weight = 0.14) in the stress cluster, and “Intolerant” (D5–D14, weight = 0.14) in the stress cluster. Finally, we found a close association between the bridge symptom “No positive” (D3) with “No wind down” (D3–D1, weight = 0.21) and “Over-react” (D3–D6, weight = 0.09) in the stress cluster and “DIF” (D3–S1, weight = 0.09) in the alexithymia cluster.

### 3.5. Results of network comparison

**FIGURE 6:**
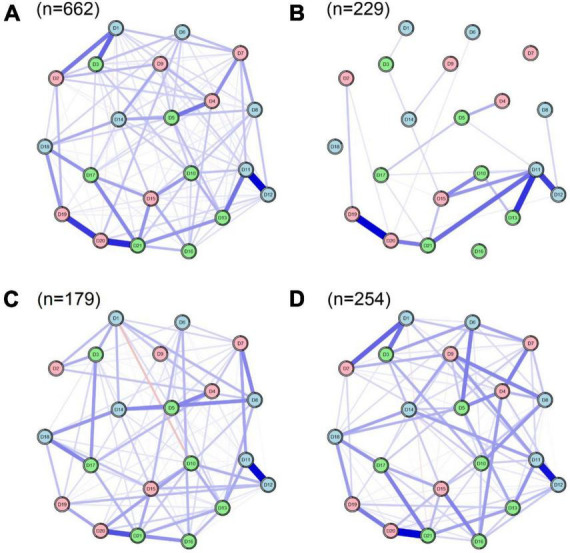
Structure of the depression, anxiety, and stress network in older adults with different degrees of alexithymia co-morbidity. **(A)** All elderly (*n* = 662). **(B)** Non-alexithymia elderly (*n* = 229). **(C)** Suspected alexithymia elderly (*n* = 179). **(D)** Alexithymia elderly (*n* = 254).

We divided the chronically co-morbid older adults into three categories based on the degree of alexithymia: the non-alexithymia group, the suspected alexithymia group, and the alexithymia group. We then constructed network structure maps of depression, anxiety, and stress for each of these three groups of older adults to test for network structure invariance, global strength invariance, and edge strength invariance, as shown in Figure 6. We found a difference in the global network structure between the alexithymia and non-alexithymia groups (*M* = 0.305, *p* = 0.013). The global strength was 9.69 for the alexithymia group and 1.64 for the non-alexithymia group, and we estimated a significant difference between the two groups (*S* = 8.046, *p* < 0.001). However, there were no significant differences in network structure between the suspected alexithymia group and the non-alexithymia group (*M* = 0.276, *p* = 0.136) or the alexithymia group (*M* = 0.206, *p* = 0.662). Using the Holm–Bonferroni correction for multiple comparisons, we found marginal differences in the network between the alexithymia and non-alexithymia groups, including “No initiative” and “Over-react” (D5–D6, *p* = 0.000), “Nervous energy” and “Worried” (D8–D9, *p* = 0.000), “Agitated” and “Meaningless” (D11–D21, *p* = 0.000), and “Scared” and “Meaningless” (D20–D21, *p* = 0.000). Finally, we found significant differences between the two groups in the global strength in all of the 21 nodes (*p* < 0.05). The strength centrality figure for each group are shown in Supplementary Figure S1.

## 4. Discussion

To the best of our knowledge, this was the first study to explore the network structure relationships between alexithymia, depression, anxiety, and stress in older adults with MCC in Chinese communities. We conducted a network analysis to identify the core and bridge symptoms in the network. We also focused on symptoms in the network with strong edge connections. Finally, we analyzed the differences in network structures between the alexithymia and non-alexithymia groups by classifying them according to the degree of alexithymia. We validated the accuracy and stability of the network, which enhanced the reliability and validity of our results (28).

Our focus on identifying core symptoms aimed to determine the main targets for psychological interventions in older adults with MCC (26). DIF tendency had the highest strength centrality in the network, indicating that it is a core node in the psychological problems of older adults with MCC from a mechanistic perspective (24). DIF, a major dimension of the alexithymia cluster, manifests as difficulties in identifying emotions in oneself or others (31). For instance, many people are often unaware of the activities happening within them and do not know if their existing feelings are sadness or anger. Previous total score-based studies (14, 45) have found that DIF scores were highest in chronically ill older adults with alexithymia, further reinforcing its central role. Core nodes can spread their effects to peripheral nodes and thus be the main targets of psychological interventions (26). Therefore, it is recommended to take effective measures to address the tendency of DIF in older adults with MCC in the communities. This may help promote improvement in other dimensions of their alexithymia, as well as alleviate symptoms of depression and anxiety.

In addition, “Meaningless,” “Agitated,” “Scared,” and “No look forward” also play central roles in the psychological problems of older adults with MCC. Among these core symptoms, the marginal strength between “Scared” and “Meaningless” ranked third in the whole network, indicating that the two core symptoms are closely related and often co-occur. Compared to young people, older adults have gone through most of life’s processes and seem to care more about the meaning of life than young people’s desire to pursue life goals (46). Older adults with MCC face multiple illnesses that make them prone to symptoms such as insomnia, irritability, anxiety, a tendency to fear the future, and a lack of expectation in life (4, 47). When these core problems are not addressed and improved, they can lead to a sense of meaninglessness in life, which triggers a chain reaction of negative psychological problems (48). Targeted interventions should be implemented for core symptoms such as “Meaningless” and “No look forward” because they serve as “target symptoms” in the psychological distress of older adults with MCC.

Predictability often represents the degree to which a node can be explained by changes in the surrounding connected nodes (43). In other words, nodes with high predictability can be controlled by controlling the neighboring nodes (44). The average predictability of the network constructed in this study was 0.51, indicating that the nodes in the network were moderately intervenable. The nodes with the highest predictability were “DIF,” “DDF,” “Meaningless,” “Agitated,” and “Scared.” This is interesting because we found that these symptoms were almost consistent with the core symptoms we identified. This implies that by controlling these core symptoms and the symptoms with the strongest connections to them, it is possible to effectively disrupt the influence of core symptoms in the network and achieve relief from psychological disorders in older adults with MCC. Specifically, “DIF” and “DDF,” as well as “Meaningless” and “Scared,” emerge as the strongest edge connections and are also identified as core nodes in the network. This further confirms the necessity for clinical healthcare professionals to intervene in these core symptoms, as they hold crucial positions within the network.

The present study identified “DIF” and “DDF” as the strongest edge connections within the alexithymia cluster, which is consistent with findings from previous network analyses in adolescent populations (49, 50). Notably, “DIF” emerged as the most predictable and strongest node in the network. Building upon these findings, we propose that targeting the “DDF” node through interventions may effectively weaken the association between “DIF” and “DDF,” subsequently mitigating the impact of “DIF” in the elderly with MCC. Previous studies have demonstrated the effectiveness of group cognitive interventions (51) and mental health programs (52) in alleviating alexithymia, yet research focusing on core symptoms of alexithymia in older adults remains limited and warrants further investigation. Furthermore, the study highlighted the robust connections between “Agitated” and “No relax” (D11–D12) and “Agitated” and “Down-hearted” (D11–D13) within the stress cluster, with “Agitated” identified as a core and predictable node in the network—excluding “Down-hearted.” Given the disease burden and psychological stress experienced by older adults with MCC, agitation and overstress are common. Previous studies have underscored the close relationship between agitation and frustration (53). Therefore, these connections deserve attention in future research. Moreover, we should not overlook the borderline link between “Heart aware” and “Scared” (D19–D20). Although the strength of “Heart aware” in the network is relatively weak (Strength = −0.46), the presence of the strongest edge with the core symptom “Scared” suggests that triggering “Heart aware” poses a high risk of activating the core symptom in the network, resulting in a global network response. Hence, older adults who frequently experience unexplained panic or arrhythmia should be mindful of their level of “Scared.”

Furthermore, the utilization of the bridge centrality index offers valuable insights into identifying bridge symptoms that play a crucial role in the development and maintenance of psychological issues among older adults with MCC (25). In our current network analysis, we identified five bridge symptoms, namely “Panic” and “Scared” from the anxiety cluster, “No wind down” from the stress cluster, “No initiative” and “No positive” from the depression cluster. These findings suggest that targeting “Panic” and “Scared” in older adults experiencing anxiety may help mitigate the risk of transmitting symptoms to other clusters, such as alexithymia, depression, or stress (25, 54). Similarly, addressing “No wind down” in the presence of stress symptoms or treating “No initiative” and “No positive” in individuals with depressive symptoms may yield similar benefits. Furthermore, we found that the bridge symptom “Panic” in the anxiety cluster was more closely associated with the depression cluster than the alexithymia and stress clusters, including feelings of meaninglessness, worthlessness, etc. This was determined by examining the edges of the bridge symptom that demonstrated the closest connections with the other three clusters. Our findings align with the diagnostic criteria presented in the Diagnostic and Statistical Manual of Mental Disorders (DSM-5) (55) and the ICD-10 (56) at the disease level. Older adults with MCC often experience varying degrees of dysfunction due to physiological degeneration and declining resilience within their bodies (57). Individuals with lower psychological resilience may develop feelings of hopelessness and helplessness (12, 47), which, when combined with the breakdown of their psychological defenses, can lead to a lack of life expectations and the emergence of symptoms such as depression and frustration. This pattern was further supported by a recent study involving older adults (53). Therefore, when older adults with MCC exhibit symptoms of anxiety, particularly feelings of “panic,” they are at an increased risk of developing depression.

As shown in Figure 5, we can visualize that the bridge symptoms “No initiative” and “No positive” in the depression cluster are between anxiety and stress symptoms. This means that, as bridge symptoms, they are more closely associated with anxiety and stress (25). Of course, relying solely on visuals to make such judgments is not convincing (25, 58). Therefore, we calculated the edge weights and found that the edges most strongly connected to “No initiative” and “No positive” were from the anxiety and stress cluster, thus reinforcing our previous judgments. The symptoms most strongly associated with them included some somatization-oriented symptoms, such as feeling tongue-tied and breathlessness in the anxiety cluster. Indeed, these symptoms are common in patients with anxiety disorders (59, 60), and our study confirms the close link between the bridging symptoms in the depression cluster and these somatization symptoms, which could be the focus of interventions in this population. In the alexithymia cluster, “DIF” had the strongest edge with the bridging symptom “No wind down” in the stress cluster and the bridging symptom “No positive” in the depression cluster. We have already mentioned several times above that “DIF” is the strongest and most predictable node in the network, thus this also needs our attention.

To test our initial hypothesis, we conducted a comparative network analysis by categorizing chronically co-morbid older adults into non-alexithymia, suspected alexithymia, and alexithymia groups. Subsequently, we constructed depression, anxiety, and stress networks for each group. Our results demonstrated that the symptom networks in the alexithymia group exhibited significantly higher global network and global strength measures compared to the non-alexithymia group, thus confirming our initial hypothesis. Consistent with previous studies, alexithymia patients are more likely to experience symptoms of depression, anxiety, and stress (9, 10, 61). Consequently, it is crucial to prioritize the mental health of individuals with alexithymia. To further investigate specific distinctions between the alexithymia and non-alexithymia groups, we examined global and local borderline differences. In comparison to the non-alexithymia group, the alexithymia group demonstrated three stronger limbic connections: “No initiative” and “Over-react” (D5–D6), “Nervous energy” and “Worried” (D8–D9), and “Scared,” and “Meaningless” (D20–D21). These symptoms spanned across depression, anxiety, and stress, indicating a higher prevalence of co-occurring psychological issues within the alexithymia group. Studies in psychopathology have previously identified impaired emotional processing in the occipital region among individuals with alexithymia (62), rendering them less responsive to emotions and more prone to employing negative emotion regulation strategies (49). This may partly explain the elevated incidence of mental health problems among older adults. Furthermore, we discovered that the non-alexithymia group exhibited stronger connections between “Agitated” and “Meaningless” (D11–D21) compared to the alexithymia group. This finding is intriguing and may serve as a focal point for interventions targeting psychological problems in the non-alexithymia group of older adults. Although the differences between the suspected alexithymia group and both the non-alexithymia and alexithymia groups did not reach statistical significance, we observed that the suspected alexithymia group exhibited network connectivity patterns closely resembling those of the alexithymia group. Therefore, it is essential not to overlook the elderly individuals in the suspected alexithymia group. Furthermore, as the Network Comparison Test is a cutting-edge method, the results obtained using this method need to be interpreted with caution, which will also need to be verified in future studies.

To the best of our knowledge, this study represents the first investigation into the relationship between alexithymia and the network structure encompassing depression, anxiety, and stress in older adults with MCC within the Chinese communities. Our findings have important implications for the prevention and intervention of psychological problems in this population. Firstly, we observed that “DIF” exhibited the highest strength and predictability within the network, indicating its central role in psychological problems among older adults. From a network perspective, effective interventions targeting DIF tendency are crucial for alleviating psychological distress in this population. Secondly, our study identified “panic,” “scared,” “No wind down,” “No initiative,” and “No positive” as bridge symptoms within the network. These bridge symptoms suggest an intertwined relationship between alexithymia and other psychological problems. Interventions focusing on these bridge symptoms may be effective in preventing or treating the comorbidity of psychological problems in chronically ill older adults. Lastly, our findings demonstrated significantly higher strength on the edges connecting “No initiative” and “Over-react” (D5–D6), “Nervous energy” and “Worried” (D8–D9), and “Scared” and “Meaningless” (D20–D21) in the alexithymia group compared to the non-alexithymia group. Targeting these specific edges through interventions may be effective in preventing and halting the progression toward alexithymia. Taken together, our study highlights the importance of addressing alexithymia and its associated network structure when designing prevention and intervention strategies for psychological problems in older adults with MCC.

## 5. Limitations

When interpreting the findings of our study, it is important to acknowledge several limitations. Firstly, we utilized the DASS-21 scale instead of the more commonly used PHQ-9 and GAD-7 scales, which may limit the comparability of our results with other studies and the generalizability of our conclusions. However, previous research has demonstrated the reliability and applicability of the DASS-21 scale in the elderly population, and the inclusion of additional symptoms in our study adds richness to the findings. Secondly, our use of cross-sectional data to construct the network prevents us from establishing causal relationships. A further investigation employing longitudinal approaches, such as cross-lagged network analysis models, is needed to examine the temporal dynamics of the core symptom “DIF” and its relationships with other variables. Additionally, the convenience sampling method used in our study may introduce selection bias, and future research should employ stratified sampling to ensure a more representative sample. Thirdly, our reliance on self-report scales for symptom assessment introduces the potential for self-report bias and may impact the accuracy of our analysis. While a combination of online and offline questionnaires allows for comprehensive participant inclusion, it also introduces additional factors that should be carefully considered. Future research should take into account the role of covariates (e.g., gender, age, and education levels) in network analyses to provide a more nuanced understanding of the relationships between symptoms. Lastly, our sample was limited to a single province in China, which may restrict the generalizability of our findings to other regions. Future studies should expand the sample, particularly among older adults in different provinces and regions, to validate the applicability and generalizability of our results at a national level. In conclusion, our study offers valuable insights into the network structure of psychological problems in older adults with MCC. However, it is important to consider these limitations and address them in future research to further enhance our understanding of the mental health of this population.

## 6. Conclusion

In conclusion, our study has provided new insights into the structure of the network between alexithymia and depression, anxiety, and stress in older adults with MCC. Our findings suggest that “DIF” is the most central node in the network, followed by “Meaningless,” “Agitated,” “Scared,” and “No look forward.” “Panic,” “Scared,” “No wind down,” “No initiative,” and “No positive” were the bridge symptoms in the network. Furthermore, we also identified differences in the network structure between the alexithymia and non-alexithymia groups. The symptoms and their interrelationships revealed in our study can provide new insights and references for the prevention and intervention of alexithymia and psychological problems in community-based elderly individuals with chronic diseases. However, the above findings should be further validated in future studies.

## Data availability statement

The datasets presented in this article are not readily available because the dataset for this study will not be made publicly available due to ethical restrictions. The dataset will be personally available if there is reasonable request. Requests to access the datasets should be directed to BS, sevenage007@163.com.

## Ethics statement

The studies involving human participants were reviewed and approved by the Jiangsu University. The patients/participants provided their written informed consent to participate in this study.

## Author contributions

BS and RC designed the study. FL, JW, XS, and QL collected the data. BS analyzed the data and wrote the original manuscript. CL and RC reviewed and edited the manuscript. All authors had contributed to the article and approved the submitted version.

## Footnotes

1
www.wjx.cn
