# Supplementary material for: The relationship between alexithymia, depression, anxiety, and stress in elderly with multiple chronic conditions in China: a network analysis
Source: Front Psychiatry. 2023 Jul 17;14:1209936. doi: 10.3389/fpsyt.2023.1209936 (PMC10389667; doi:10.3389/fpsyt.2023.1209936)
Supplement: Supplementary file 1 [file Data_Sheet_1.docx]

**Supplementary Material**

Table S1. Correlation matrix of the TAS-20 and DASS-21.

Figure S1. Comparison of network centrality indices between, non-alexithymia, suspected alexithymia, and alexithymia.

Table S1. Correlation matrix of the TAS-20 and DASS-21.

|  | D1 | D2 | D3 | D4 | D5 | D6 | D7 | D8 | D9 | D10 | D11 | D12 | D13 | D14 | D15 | D16 | D17 | D18 | D19 | D20 | D21 | S1 | B1 | W1 |
| --- | --- | --- | --- | --- | --- | --- | --- | --- | --- | --- | --- | --- | --- | --- | --- | --- | --- | --- | --- | --- | --- | --- | --- | --- |
| D1 |  |  |  |  |  |  |  |  |  |  |  |  |  |  |  |  |  |  |  |  |  |  |  |  |
| D2 | 0.19 |  |  |  |  |  |  |  |  |  |  |  |  |  |  |  |  |  |  |  |  |  |  |  |
| D3 | 0.21 | 0.05 |  |  |  |  |  |  |  |  |  |  |  |  |  |  |  |  |  |  |  |  |  |  |
| D4 | 0.04 | 0.06 | 0.06 |  |  |  |  |  |  |  |  |  |  |  |  |  |  |  |  |  |  |  |  |  |
| D5 | 0.03 | 0.00 | 0.02 | 0.21 |  |  |  |  |  |  |  |  |  |  |  |  |  |  |  |  |  |  |  |  |
| D6 | 0.00 | 0.07 | 0.09 | 0.00 | 0.07 |  |  |  |  |  |  |  |  |  |  |  |  |  |  |  |  |  |  |  |
| D7 | 0.09 | 0.00 | 0.05 | 0.17 | 0.04 | 0.10 |  |  |  |  |  |  |  |  |  |  |  |  |  |  |  |  |  |  |
| D8 | 0.06 | 0.02 | 0.00 | 0.04 | 0.03 | 0.02 | 0.14 |  |  |  |  |  |  |  |  |  |  |  |  |  |  |  |  |  |
| D9 | 0.01 | 0.09 | 0.01 | 0.10 | 0.07 | 0.05 | 0.02 | 0.03 |  |  |  |  |  |  |  |  |  |  |  |  |  |  |  |  |
| D10 | -0.01 | 0.00 | 0.07 | 0.02 | 0.08 | 0.00 | 0.01 | 0.09 | 0.02 |  |  |  |  |  |  |  |  |  |  |  |  |  |  |  |
| D11 | 0.04 | 0.00 | 0.02 | 0.03 | 0.03 | 0.00 | 0.10 | 0.00 | 0.07 | 0.09 |  |  |  |  |  |  |  |  |  |  |  |  |  |  |
| D12 | 0.00 | 0.02 | 0.02 | 0.02 | 0.00 | 0.05 | 0.00 | 0.09 | 0.00 | 0.02 | 0.41 |  |  |  |  |  |  |  |  |  |  |  |  |  |
| D13 | 0.00 | 0.00 | 0.02 | 0.01 | 0.10 | 0.00 | 0.03 | 0.06 | 0.04 | 0.12 | 0.22 | 0.06 |  |  |  |  |  |  |  |  |  |  |  |  |
| D14 | 0.05 | 0.02 | 0.04 | 0.00 | 0.14 | 0.05 | 0.06 | 0.10 | 0.13 | 0.08 | 0.07 | 0.01 | 0.00 |  |  |  |  |  |  |  |  |  |  |  |
| D15 | 0.00 | 0.00 | 0.05 | 0.02 | 0.00 | 0.02 | 0.00 | 0.07 | 0.00 | 0.16 | 0.04 | 0.04 | 0.00 | 0.09 |  |  |  |  |  |  |  |  |  |  |
| D16 | 0.03 | 0.00 | 0.00 | 0.08 | 0.01 | 0.02 | 0.01 | 0.07 | 0.00 | 0.06 | 0.00 | 0.05 | 0.05 | 0.00 | 0.13 |  |  |  |  |  |  |  |  |  |
| D17 | 0.00 | 0.00 | 0.06 | 0.06 | 0.06 | 0.00 | 0.02 | -0.04 | 0.02 | 0.00 | 0.00 | 0.00 | 0.04 | 0.07 | 0.14 | 0.03 |  |  |  |  |  |  |  |  |
| D18 | 0.02 | 0.06 | 0.00 | 0.00 | 0.07 | 0.07 | 0.00 | 0.00 | 0.00 | 0.02 | 0.00 | 0.01 | 0.01 | 0.13 | 0.01 | 0.00 | 0.17 |  |  |  |  |  |  |  |
| D19 | 0.00 | 0.10 | 0.00 | 0.00 | 0.00 | 0.01 | 0.00 | 0.03 | 0.04 | 0.00 | 0.00 | 0.02 | 0.03 | 0.01 | 0.04 | 0.00 | 0.00 | 0.17 |  |  |  |  |  |  |
| D20 | -0.04 | 0.03 | 0.00 | 0.00 | 0.00 | 0.11 | 0.00 | 0.02 | 0.00 | 0.03 | 0.00 | 0.07 | 0.02 | 0.00 | 0.01 | 0.09 | 0.00 | 0.03 | 0.31 |  |  |  |  |  |
| D21 | 0.00 | -0.01 | 0.04 | 0.00 | 0.00 | -0.02 | 0.00 | 0.00 | 0.00 | 0.08 | 0.00 | 0.03 | 0.17 | 0.00 | 0.17 | 0.11 | 0.19 | 0.01 | 0.00 | 0.35 |  |  |  |  |
| S1 | 0.12 | 0.05 | 0.09 | 0.07 | 0.02 | 0.05 | 0.00 | 0.00 | 0.08 | 0.02 | 0.00 | 0.00 | 0.00 | 0.00 | 0.00 | 0.03 | 0.00 | 0.00 | 0.00 | 0.00 | 0.02 |  |  |  |
| B1 | 0.00 | 0.03 | 0.00 | 0.00 | 0.01 | 0.00 | 0.03 | 0.00 | 0.00 | 0.00 | 0.00 | 0.00 | 0.00 | 0.00 | 0.02 | 0.00 | 0.00 | 0.00 | 0.06 | 0.00 | 0.00 | 0.64 |  |  |
| W1 | 0.02 | 0.00 | 0.02 | 0.00 | 0.00 | 0.00 | 0.00 | 0.00 | 0.05 | 0.09 | 0.06 | 0.00 | 0.00 | 0.00 | 0.02 | -0.02 | 0.03 | 0.00 | -0.08 | -0.06 | 0.00 | 0.14 | 0.19 |  |

D1, No wind down; D2, Dry mouth; D3, No positive; D4, Breath difficult; D5, No initiative; D6. Over-react; D7, Trembling; D8, Nervous energy; D9, Worried; D10, No look forward; D11, Agitated; D12, No relax; D13, Down-hearted; D14, Intolerant; D15, Panic; D16, Not enthusiastic; D17, Worthless; D18, Touchy; D19, Heart aware; D20, Scared; D21, Meaningless; S1, DIF; B1, DDF; W1, EOTS.


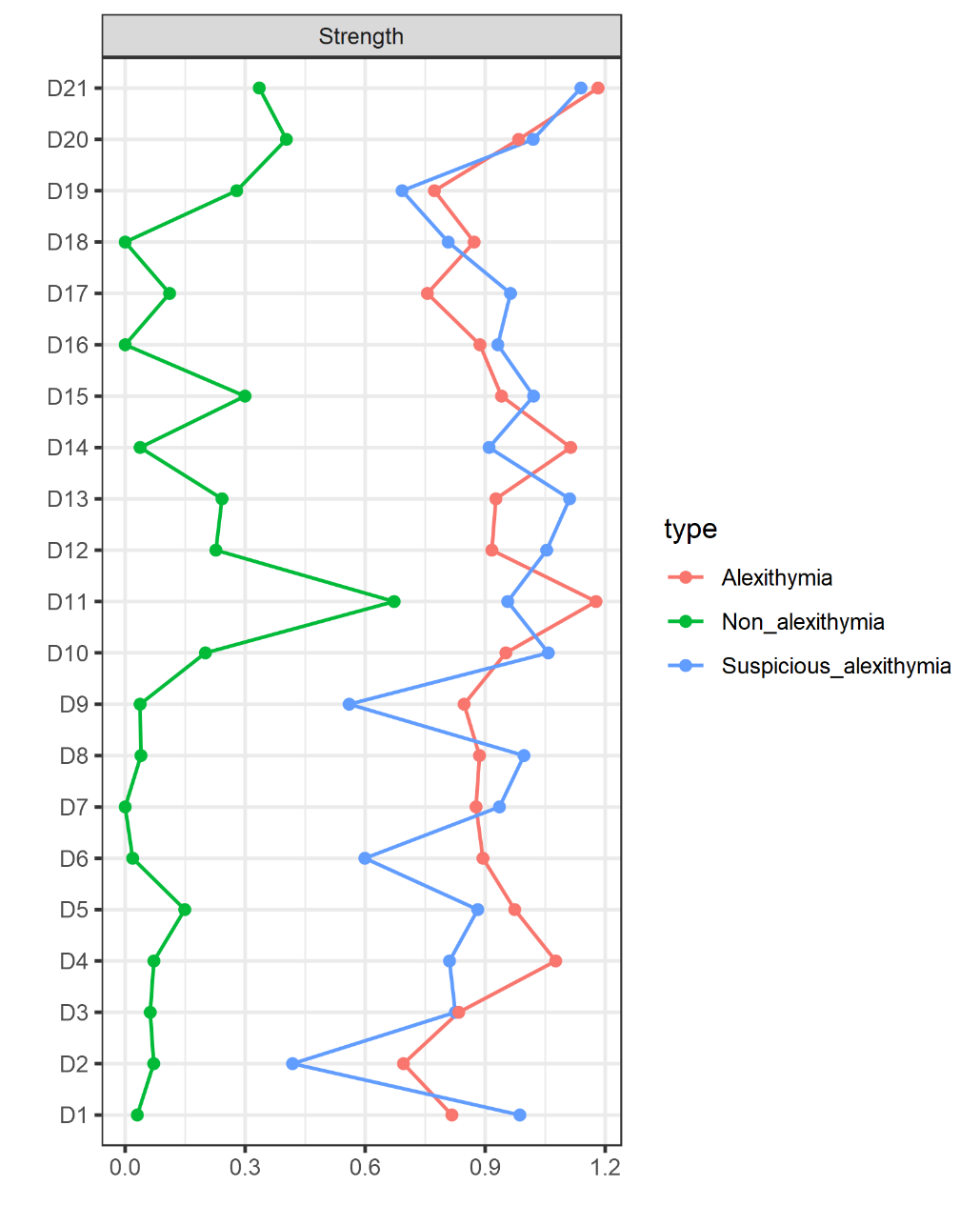


Figure S1. Comparison of network centrality indices between, non-alexithymia, suspected alexithymia, and alexithymia.

D1, No wind down; D2, Dry mouth; D3, No positive; D4, Breath difficult; D5, No initiative; D6. Over-react; D7, Trembling; D8, Nervous energy; D9, Worried; D10, No look forward; D11, Agitated; D12, No relax; D13, Down-hearted; D14, Intolerant; D15, Panic; D16, Not enthusiastic; D17, Worthless; D18, Touchy; D19, Heart aware; D20, Scared; D21, Meaningless;
